# *Emblica officinalis* extract downregulates pro-angiogenic molecules *via* upregulation of cellular and exosomal miR-375 in human ovarian cancer cells

**DOI:** 10.18632/oncotarget.8966

**Published:** 2016-04-25

**Authors:** Alok De, Benjamin Powers, Archana De, Jianping Zhou, Siddarth Sharma, Peter Van Veldhuizen, Ajay Bansal, Ramratan Sharma, Mukut Sharma

**Affiliations:** ^1^ Kansas City VA Medical Center and Midwest Biomedical Research Foundation, Kansas City, MO 64128, United States

**Keywords:** ovarian cancer, Emblica officinalis, microRNA-375, IGF1R, exosomes

## Abstract

Ovarian cancer (OC) is highly resistant to current treatment strategies based on a combination of surgery, chemotherapy and radiation therapy. We have recently demonstrated the anti-neoplastic effect of Amla extract (*Emblica officinalis*, AE) on OC cells *in vitro* and *in vivo*. We hypothesized that AE attenuates growth of OC through microRNA (miR)-regulated mechanism(s). The inhibitory effect of AE on proliferation, migration and invasiveness (P≤0.001) of SKOV3 cells and >90% attenuation of tumor growth in a xenograft mouse model suggested multiple targets. RT-qPCR analysis of microRNAs associated with OC showed a >2,000-fold increase in the expression of miR-375 in AE-treated SKOV3 cells that was blocked by an exogenous miR-375 inhibitor (P≤0.001). AE also decreased the gene and protein expression of IGF1R, a target of miR-375 (P≤0.001), and SNAIL1 (P≤0.002), an EMT-associated transcription factor that represses E-cadherin expression (P≤0.003). AE increased E-cadherin expression (P≤0.001). Treatment of SKOV3 cells with AE resulted in increased miR-375 in exosomes in the medium (P≤0.01). Finally, AE significantly decreased the expression of IGF1R and SNAIL1 proteins during attenuation of SKOV3-derived xenograft tumor. Together, these results show that AE modulates cancer cells and the tumor microenvironment *via* activation of miR-375 and by targeting IGF1R and SNAIL1 in OC cells.

## INTRODUCTION

Ovarian cancer (OC) is a leading cause of female mortality [1]. Globally, approximately 190,000 new cases of OC are diagnosed each year [2]. In United State approximately 22,000 women were diagnosed with OC, and 15,000 women died from OC in 2014 alone [1]. Early diagnosis of OC is often missed and aggressive intervention at late stages is generally unsuccessful. Current treatment of OC relies on a combination of surgery, chemotherapy and radiation that yields poor results with a five year survival rate of about 30% [3]. Anticancer drugs are often highly toxic and contribute to morbidity and poor outcome [4]. There is an ongoing effort to identify novel therapeutic agents to target multiple cellular processes for a comprehensive inhibition of tumor growth and metastasis.

Plant-derived products with anti-tumor effects and low toxicity [4, 5] are useful in developing cost effective alternative or adjunct cancer therapies [4]. Amla (*Emblica officinalis*) is an ingredient of preparations used for treating cancer [5] in the ayurvedic and Middle Eastern pharmacopeia. The water extract of Amla (fruit, leaves, bark, root) has antioxidant, antihypertensive, anti-atherogenic, anti-inflammatory and anti-neoplastic properties. Amla extract (AE) inhibits proliferation of a variety of cancer cells including OC cells *in vitro* as well as tumor growth *in vivo* [5, 6]. Recently we reported that AE reduces cell proliferation, tumor growth, angiogenesis in OVCAR3 cells [6]. The mechanism of its anti-tumorigenic effect is not known.

MicroRNAs (miRs) are versatile small non-coding single-stranded RNAs that control gene expression post-transcriptionally by binding to complementary sequences in the 3′ untranslated region (UTR) of target messenger RNA (mRNA) resulting in either degradation of the transcript or inhibition of translation [7]. Recent reports show that several miRs are associated with OC [8]. One or more target proteins can be regulated by one miR and, one or more miRs may target one protein. The pro- or anti-oncogenic effect of miRs is determined by the target protein through mir-miRNA interaction [9]. Signature miRs are being explored as molecular diagnostic markers of disease as well as targets and agents for specific intervention [10]. MicroRNAs are also present in circulation suggesting their likely role in intercellular communication and potentially in disease mechanisms.

The metastatic and resistant nature of OC implies its ability for transformation and migration that may significantly affect the interaction between cancer cells and the microenvironment [11]. Exosomes are being explored as effective mediators of communication between cells and their environment [12]. Exosomes are small secreted membrane vesicles (30-100 nm) that contain miRs as well as a variety of cell surface and cytoplasmic proteins as their cargo [13]. The effect of AE on exosomes derived from OC cells is not known.

We hypothesized that the anti-cancer effect of AE on OC cells is mediated through miRs. *In vitro* experiments using SKOV3 cells show that AE upregulated miR-375 and adhesion protein E-cadherin but down regulated insulin-like growth factor 1 receptor (IGF1R) and epithelial-mesenchymal transition (EMT) factor SNAIL1. Additional experiments showed that total exosomal protein and miR-375 secreted with exosomes were upregulated following AE treatment. Results show that AE has anti-proliferative, anti-migratory and anti-invasive effects on SKOV3 ovarian cancer cells *in vitro*. Finally, *in vivo* experiments show AE attenuated the growth of the xenograft and expression of IGF1R and SNAIL1 while increasing the expression of E-cadherin in the tumor. Results of *in vitro* and *in vivo* experiments to characterize a potential role of miR-375 in the anti-ovarian cancer effects of AE are presented.

## RESULTS

### AE inhibits SKOV3 cells proliferation/viability

**Figure 1:**
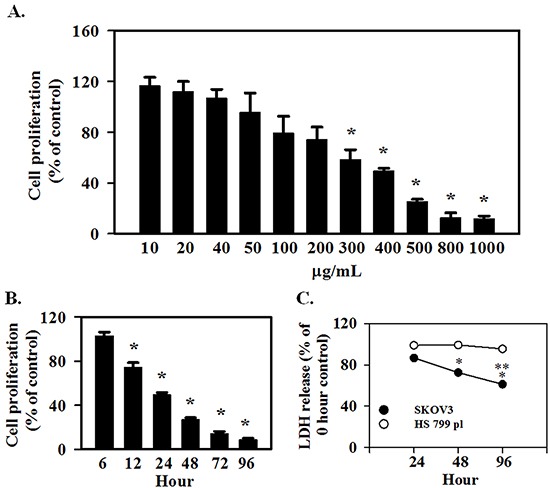
*E. officinalis* (Amla) extract (AE) inhibits cell proliferation in ovarian cancer cells SKOV3 and HS 799.Pl placental cells were grown for 2 days in DMEM as described under Materials and Methods. **A.** To determine the effect of AE concentration on proliferation, SKOV3 cells were treated with 10-1000 μg/ml AE for 24 hours. AE decreased the proliferation of SKOV3 cells in a dose-dependent manner. * indicates P≤0.05 *vs.* the vehicle-treated control group. **B.** To determine the temporal effect of AE on proliferation, SKOV3 cells were treated with 400 μg/ml of AE for 6-96 hours. * indicates P<0.05 *vs.* the vehicle-treated control group. **C.** To determine the cytotoxicity of AE, SKOV3 and HS 700.Pl placental cells were treated with 400 μg/ml of AE for 24, 48 and 96 h. Results are presented as percent of untreated control cells at each time point. ^*^ indicates P<0.05 *vs.* 24 hour, ^**^ indicates P≤0.05 *vs.* values at 48 hour. All results are presented as Means ±SEM from 6 independent observations.

SKOV3 cells are a highly aggressive OC cell line and an anti-proliferative effect of AE would provide strong validation of our previous observations based on using OVCAR3 cells [14]. SKOV3 cells were treated with varying concentrations of AE (0-1000 μg/ml) for 24 h time period and used for MTT assays. Figure 1A shows that AE inhibited the proliferation of SKOV3 cells in a concentration-dependent manner. Cell proliferation/viability was not affected by low concentrations (10-200 μg/ml) of AE. However, cell proliferation/viability was significantly inhibited at AE concentrations 300–1000 μg/mL with the IC_50_ at 400 μg/mL. AE was used at this dose (400 μg/mL) for other experiments. Figure 1B shows that AE time dependently caused significant inhibition of SKOV3 cells. At 12 hour, AE caused significant inhibition of cell proliferation/viability (P=0.007), however inhibition of cell proliferation was only about 30% that of control.

### AE does not cause cytotoxicity in normal placental cells

To determine the cytotoxic effect of AE, SKOV3 and Hs 799.Pl cells were treated with 400 μg/ml AE for 24 h. Cytotoxicity of AE on SKOV3 and Hs 799.Pl was determined by measuring LDH released into the culture medium as a marker of dead cells. Figure 1C shows that AE did not cause cytotoxic effect on Hs 799.Pl cells up to 96 h compared with 0 h. However, significant cytotoxic effects were noted in SKOV3 cells (P=0.002).

### AE inhibits OC cells migration and invasion

**Figure 2:**
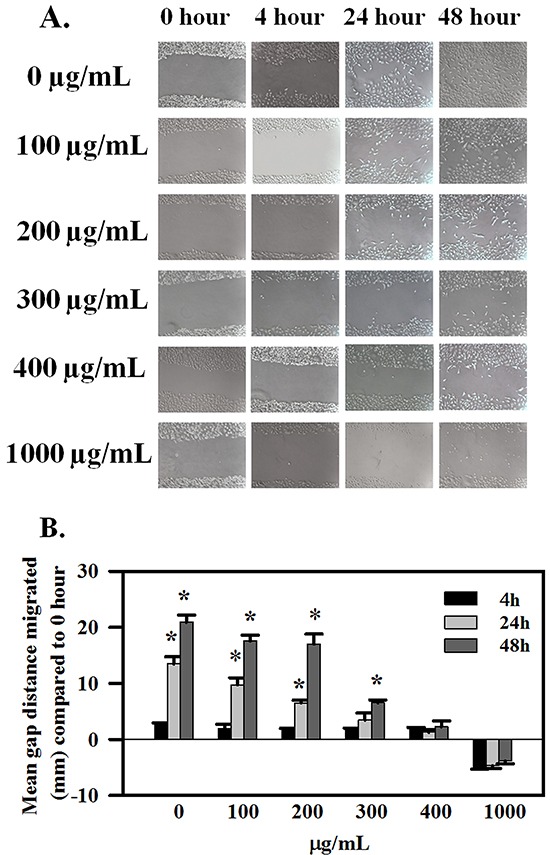
AE inhibits wound-healing in ovarian cancer cells in a time- and dose-dependent manner SKOV3 cells at 90% confluency were scratch-wounded and treated with 100-1000 μg/ml AE and further incubated for 48 h. **A.** Representative images showing the inhibitory effect of AE on wound-healing migration of SKOV3 cells captured at 0, 4, 24 and 48 h time points, respectively. **B.** Quantitative analysis of the results presented under (**A**). Gap distances were measured and normalized using an untreated control. Data are shown as Means ± SEM (n=3 separate experiments). * indicates P≤0.001 compared with 0 hour.

A potential effect of AE in OC metastasis on migration and invasion was studied using SKOV3 cells. Figure 2A presents results of the scratch wound healing assay. Treatment with AE revealed significant dose- and time-dependent inhibitory effect of AE on the migration of SKOV3 cells into the wound area. Only 1000 μg/mL of AE showed significant inhibition of migration at 4 h. Three hundred and 400 μg/mL of AE inhibited SKOV3 cells wound healing at 24 hours and 48 hours. Two hundred of AE inhibited SKOV3 cells wound healing after 24 hours of treatment but that effect was not significant (Figure 2A and 2B). A comparison of relative gap distances after treatment with AE is shown in Figure 2B. Overall, AE (≥300μg/mL) significantly attenuated the rate of wound healing (measured as relative gap distance in millimeters) in SKOV3 cells compared to untreated cells (P≤0.001).

**Figure 3:**
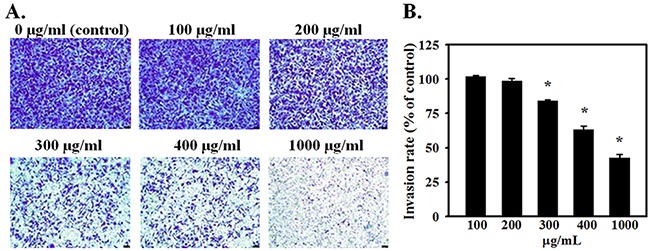
AE suppresses the invasiveness of ovarian cancer cells in a dose-dependent manner SKOV3 cells were seeded onto the transwell insert consisting of 8 μm pore-size filters coated with Matrigel basement membrane matrix. Inserts were placed in wells containing 0, 100, 200, 300, 400 or 1000 μg/ml of AE in the medium and incubated for 24 h. **A.** Migrated cells were stained with crystal violet and photographed. Representative images (×200) show SKOV3 cells that crossed the matrigel coated membrane indicating invasiveness. **B.** Quantitative results of crystal violet released from migrated cells using spectrophotometry at 590 nm. Data are shown as Means ± SEM. from 4 independent observations. * indicates P<0.05 *vs.* the vehicle-treated control group. Bar=25 μM.

Migration/invasion in Transwells was followed by crystal violet staining and imaging the cells that migrated across the Transwell membrane, and by quantitation of the intensity of stain. Figure 3A shows crystal violet-stained migrated cells and Figure 3B shows the optical density of crystal violet taken up by stained cells. AE treatment dose dependently reduced migration/invasion of SKOV3 cells (P≤0.001). Collectively, these results indicate that AE inhibits both the migration and invasion abilities of SKOV3 cells.

### AE increases the expression of miR-375 in SKOV3 cells

**Figure 4:**
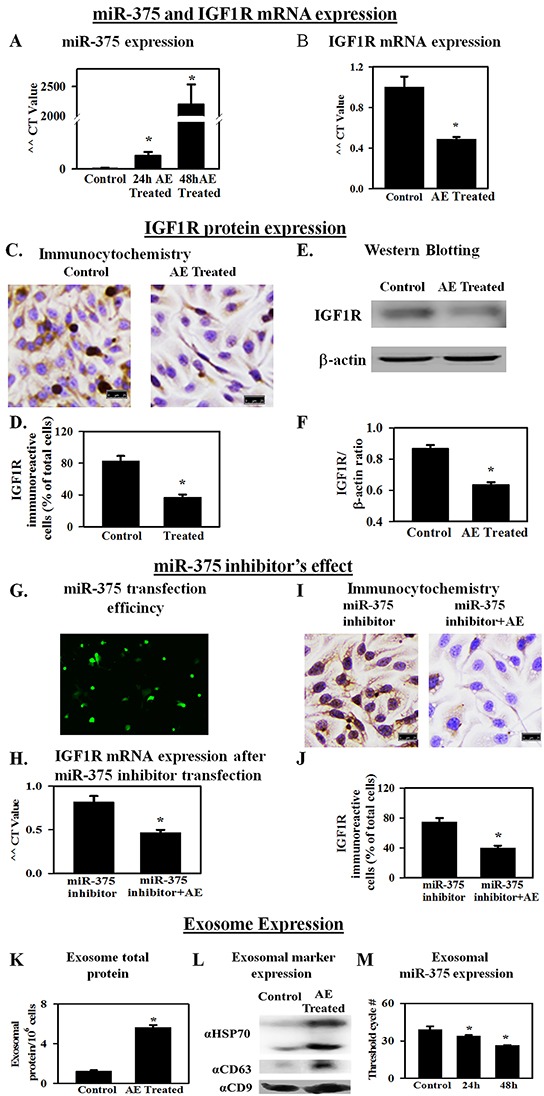
AE increases the expression of miR-375 in ovarian cancer cells as well as in exosomes in the medium IGF1R, a receptor for IGF, is associated with proliferation. miR-375 blocks the expression of IGF1R. **A.** RT-qPCR results showing a >2000-fold increase in miR-375 gene expression (48 h) in SKOV3 cells treated with AE compared to untreated control cells. **B.** RT-qPCR results (fold change) show that AE reduces IGF1R gene expression in SKOV3 cells at 24 h. **C.** Immunocytochemistry results show that AE (400 μg/ml) attenuates IGF1R protein expression in SKOV3 cells at 24 h. **D.** Bar graph shows immunopositive cells as percent of total number of cells counted. **E.** A representative photograph of SDS-PAGE Western blot analysis of the expression of IGF1R protein in SKOV3 cells treated with AE (400 μg/mL, 24 h). β -actin was used as loading control. **F.** Bar graph presents IGF1R/β -actin densitometry ratios obtained from 4 independent experiments. **G.** Representative image of transfected fluorescent anti-hsa-miR-375 miScript miRNA inhibitor in SKOV3 cells at 24 hour. **H.** RT-qPCR results (fold change) show that AE blocks the effect of transfected miR-375 inhibitor on IGF1R gene expression in SKOV3 cells at 24 h. **I.** Immunostaining for IGF1R in SKOV3 cells transfected with anti-hsa-miR-375 miScript miRNA with (400 μg/mL) and without AE. **J.** Bar graph shows IGF1R immuno-positive cells as percent of total cells. **K.** Total exosomal protein in control and *E. officinalis*-treated SKOV3 cells (48 h). **L.** Exosomal marker protein expression in control and *E. officinalis* treated SKOV3 cells (48 h) culture media. **M.** MiR-375 expression in exosomes of control and *E. officinalis* treated SKOV3 cells (48 h). Values are Means ± SEM (n=4), *, P<0.05 compared with control. Bar=25 μm.

MicroRNAs are involved in tumorigenesis and altered expression of several micro RNAs in OC has been reported [8]. Expression of candidate miRs that are upregulated (miR-27a and miR-195a) or down regulated (miR-10b, miR-375, miR-let7a, miR-let7c, miR-146a) in OC was assessed using RT-qPCR. SKOV3 cells treated with AE were analyzed for these miRs. Table 1 shows changes in the expression of miR-27a, miR-195a, miR-10b, miR-375, miR-let7a, miR-let7c, miR-146a. With variable low effect on other miRs analyzed, AE treatment caused a >14-fold and >2000-fold increase in the expression of miR-375 at 24 h and 48 h, respectively (Figure 4A). These results suggest that AE strongly induces the expression of miR-375 in OC cells.

**Table 1:** Fold changes in miRNAs after *E. officinalis* treatment

Micro RNA	AE-induced fold-change compared to untreated SKOV3 cells (control)	
	24 hours	48 hours
miR-27a	0.80±0.18	1.31±0.27
miR-195	6.36±0.14	1.80±0.07
miR-let7a	4.67±0.21	0.74±0.08
miR-let7c	0.77±0.24	4.11±0.12
miR-10b	1.96±0.37	3.30±0.15
miR-146a	4.21±0.10	3.37±0.13
miR-375	14.59±3.54	2207.44±328.06

Several microRNAs are associated with resistant OC. The effect of AE on the levels of seven microRNAs in SKOV3 cells was evaluated. Cells were incubated with AE (400 μg/mL) for 24 or 48 h. With a modest increase at 24 h, levels of miR-375 increased by 2207-fold at 48 h. Other microRNAs had relatively less effect at either time point.

### AE down regulates the expression of IGF1R in SKOV3 cells

IGF1R, a receptor for insulin-like growth factor, is regulated by miR-375 [15]. Previous experiments showed that AE induces a significant increase in the expression of miR-375 in SKOV3 cells. Therefore, the effect of AE on the gene expression of IGF1R was determined using RT-qPCR and on protein expression using immunocytochemistry and Western blotting. Results showed significantly decreased expression of IGF1R mRNA in AE-treated SKOV3cells at 24 h (P=0.007; Figure 4B) and protein expression (P≤0.001; Figure 4C–4F). SKOV3 cells were transfected with miR-375 inhibitor for 24 h. Fluorescence microscopy at 48 h revealed an even distribution of miR-375 inhibitor in SKOV3 cells (Figure 4G) with approximately 70% transfection efficiency. IGF1R mRNA in control and transfected groups was not significantly different. Addition of AE (24 h) to cells transfected cells with miR-375 inhibitor caused a significant decrease in mRNA (P=0.014; Figure 4H) and protein (P≤0.001; Figure 4I and 4J) expression of IGF1R. These results suggest a role of miR-375 in angiogenesis *via* downregulation of IGF1R since IGF1R upregulates angiogenesis [16].

### AE upregulates miR-375 in exosomes from SKOV3 cells

Exosomes are nanometer range vesicular particles laden with protein and RNA cargo [17]. Exosomes are considered as mediators of cellular communication through proteins and RNA [18]. Exosomal miRs are considered to play a role in disease progression. Exosomal proteins may initiate intracellular signaling in target cells in cancer [19]. Therefore, changes in the expression of exosomal protein and miR-375 after AE treatment were determined. Treatment of SKOV3 cells with AE resulted in a 5-6 fold increase in total exosomal protein at 48 h compared to control (P≤ 0.01). Control SKOV3 cells released 1.22 ± 0.11 μg exosomal protein/10^6^ cells/48 h and AE-treated cells released 5.66±0.22 μg exosomal protein/10^6^ cells/48 h. Results are average of 4 individual experiments using ~300 × 10^6^ cells each (Figure 4K). The exosomal marker proteins CD9, CD63 and HSP70 were detected in the exosomes released from SKOV3 cells in culture media (Figure 4L). The expression of these marker proteins was higher in the exosomes obtained from E. officinalis treated SKOV3 culture media (Figure 4L).

Incubation of SKOV3 cells with AE resulted in increased exosomal miR-375 transcripts compared to untreated SKOV3 cells (N= 4, P≤ 0.001). This finding was also indicated by decrease in threshold cycle (C_T_) numbers. Thus, the C_T_ number for detecting miR-375 was 39.12±2.56, 33.97±0.76 and 26.44±0.25 in untreated control and cells treated with AE for 24 h and 48 h, respectively (Figure 4L).

### AE decreases expression of metastasis-associated transcription factor SNAIL1 in SKOV3 cells

**Figure 5:**
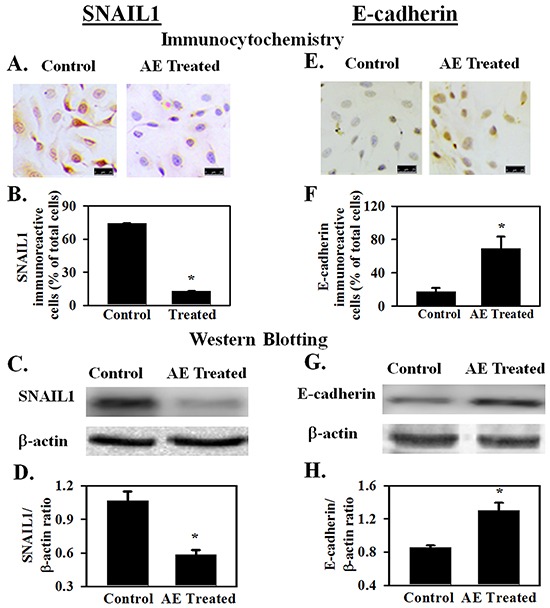
AE downregulates the expression of SNAIL1 and increases the expression of E-cadherin in SKOV3 cells SNAIL1 is a transfection factor that downregulates E-cadherin expression and promotes epithelial-mesenchymal transformation. SKOV3 cells were treated with AE (400 μg/ml) for 24 h and SNAIL1 and E-cadherin expression were studied. **A, B.** Immunocytochemical analysis shows that SNAIL1 positive cells (as percent of total cells) decreased after AE treatment. **C, D.** Western blot analysis shows that expression of SNAIL1 protein decreased in AE treated cells image and IGF1R/β-actin densitometry ratios. **E, F.** Immunocytochemical analysis shows increased number of E-cadherin positive cells (as percent of total number of cells) after AE treatment. **G, H.** Western blot analysis shows that E-cadherin protein expression increased in AE-treated cells. Representative blot image is shown along with bar graph showing IGF1R/β-actin densitometry ratios. Results are presented as Means±SEM *, P<0.05 *vs.* control, n=4 experiments. Insert bar=25 μM. β -actin was used as the loading control for Western blot analyses.

Micro RNAs are reported to directly target transcription factors that regulate epithelial mesenchymal transformation [20, 21]. Key transcription factors in this process include members of the SNAIL family that suppress epithelial markers and promote expression of mesenchymal markers [22]. SNAIL1 is a transcription factor that represses adhesion molecules such as E-cadherin [23]. SNAIL1 plays a critical role in tumor growth and metastasis of ovarian carcinoma [24]. SNAIL1 also down regulates miR-375 [25]. Effect of AE on SNAIL1 is not known. Earlier experiments showed that AE attenuates migration and invasiveness of SKOV3 cells. Therefore, we hypothesized that AE down regulates SNAIL1 expression. Results summarized in Figure 5A–5D show that AE treatment significantly reduced the expression of SNAIL1 in SKOV3 cells. Immunocytochemical images in Figure 5A and quantitative analysis in Figure 5B indicate significant decrease in SNAIL1 protein expression in AE-treated SKOV3 cells (P≤0.001). Western blot analysis revealed a significant decrease in SNAIL1 protein expression in SKOV3 cells following AE treatment (P≤0.001, Figure 5C and 5D).

### AE upregulates E-cadherin expression

SNAIL1 is a direct repressor of E-cadherin expression during development and tumorigenesis [26]. E-cadherin is considered to be a suppressor of tumor invasiveness [27]. Earlier experiments showed that AE down regulates SNAIL1 expression and suppresses migration and invasiveness of SKOV3 cells. Therefore, the effect of AE on E-cadherin protein expression was studied in AE-treated SKOV3 cells. Results summarized in Figures 5E–5H show that AE treatment significantly increased (P=0.014) the expression of E-cadherin in SKOV3 cells. Figure 5E and 5F show increased expression of E-cadherin after AE treatment using immunocytochemical techniques (P=0.014). Figures 5G and 5H show increased expression of E-cadherin after AE treatment using Western blot technique (P=0.003). These results suggest that AE modulates EMT through inhibition of SNAIL1 and activation of E-cadherin in SKOV3 cells.

### AE inhibits tumor growth and IGF1R and SNAIL1 expression, but increases E-cadherin expression in SKOV3 xenograft tumors in athymic nude mice

**Figure 6:**
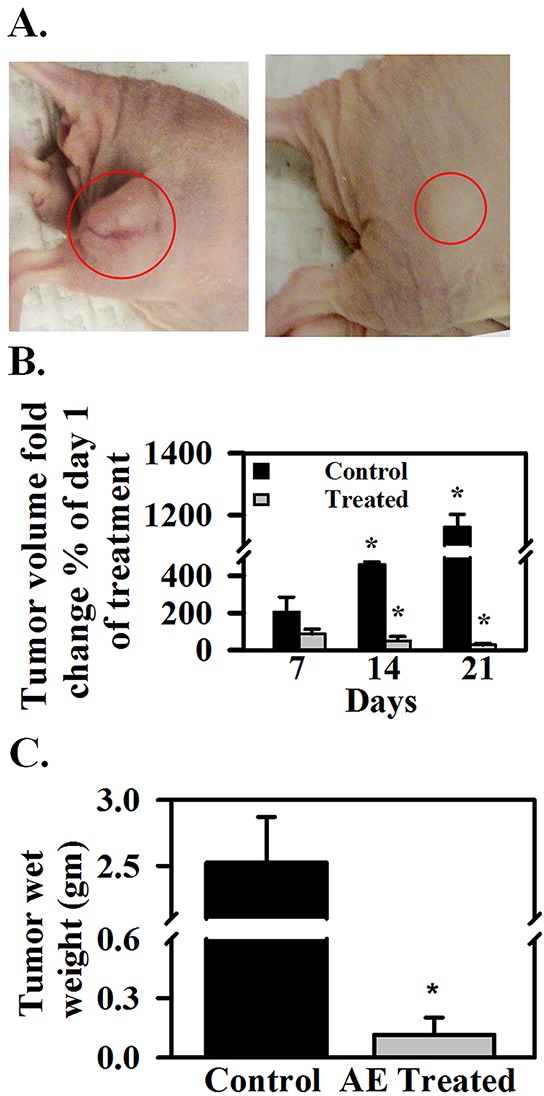
AE reduces SKOV3 xenograft tumor growth in athymic nude mice **A.** Nude mice bearing tumors, left- control, right- treated with AE in drinking water. **B.** Tumor volume on day 7, 14 and 21 as percent of day 0. **C.** tumor wet weight in grams on day 28. The values are means ± SEM. of 5 individual mice.

**Figure 7:**
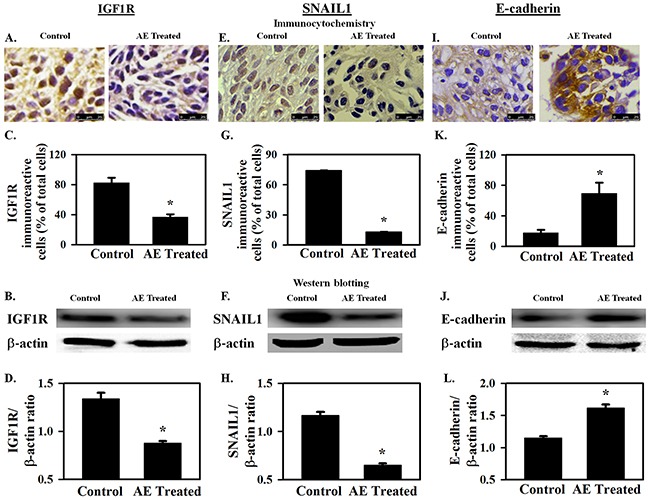
AE downregulates IGF1R and SNAIL1 protein expression and increases E-cadherin protein expression in SKOV3 xenograft tumors **A–D.** IGF1R protein expression. A, B. Immuno-cytochemical analysis shows decreased expression of IGF1R in xenografts from AE-treated mice. Bar graph shows the percentage of IGF1R immuno-reactive cells. C, D. Representative Western blot image showing decreased expression of IGF1R. Densitometry ratios of IGF1R/β -actin are shown. **E–H.** SNAIL1 protein expression. E, F. Decreased immuno-expression of SNAIL1 is observed in xenograft tumors after AE treatment. Bar graph shows SNAIL1 immuno-reactive cells as percent of total number of cells. G, H. Representative Western blot image showing decreased expression of SNAIL1. SNAIL1/β -actin densitometry ratios presented. **I–L.** E-cadherin protein expression. I, J. Image showing increased immuno-expression of E-cadherin in xenograft tumors after treatment with AE. Percentage of E-cadherin immuno-positive cells in control and AE treated groups is compared. K, L. Representative Western blot image shows increased expression of E-cadherin in AE treated group. Densitometric ratios of E-cadherin/β -actin are presented. All results were obtained using tumor tissues from 5 mice in each group. Values are Means ± SEM, *, P≤0.05 compared with control group. Bar=25 μm.

*In vivo* effects of AE on tumor growth and on miR-375 target IGF1R (promotes proliferation and growth), SNAIL1 (promotes EMT) and E-cadherin (opposes EMT) were assessed to confirm *in vitro* findings. AE treatment did not cause any toxicity as demonstrated by a lack of apparent changes in liver, spleen (appearance, gross morphology), body weight, food and water intake, grooming behavior and mobility (data not shown). Figure 6A shows attenuation of tumor growth in the AE-treated mice compared to untreated control with significant differences in tumor size at 3 weeks of treatment (Figure 6B; P=0.001). Mice were sacrificed 27 days after inoculation and tumors were excised. Tumor size and wet weight were significantly reduced in AE treated *vs.* control mice (Figure 6C; P=0.001). Tumor tissue was analyzed using immunohistochemistry and Western blotting. AE treatment significantly reduced the number of IGF1R positive cells (P≤0.001) (Figure 7A–7B) and also IGF1R protein levels (P=0.001) (Figure 7C–7D). SNAIL1 expression was significantly reduced as indicated by the count of SNAIL1 positive cell as percent of total number of cells (Figures 7E, 7F) as well as by SDS-PAGE-Western blot analysis of SNAIL1 protein in tumor xenograft (Figure 7G, 7H). E-cadherin expression was markedly enhanced in xenografts from AE-treated mice compared to controls as suggested by immunopositive cells in the immunohistochemical analysis (Figures 7I, 7J) as well as the intensity of protein band observed by SDS-PAGE-Western blot analysis (Figures 7K, 7L). Consistent with our *in vitro* findings, *in vivo* results demonstrate that AE treatment activates E-cadherin in xenograft ovarian tumors.

### AE decreases the expression of AKTP in SKOV3 cells

**Figure 8:**
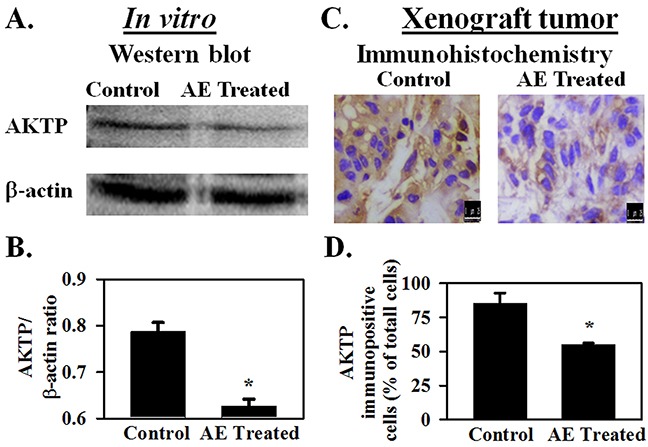
AE inhibits AKTP protein expression in ovarian cancer cells SKOV3 cells were treated with AE (400 μg/mL) for 24 h and AKTP expression was analyzed by immunostaining and Western blotting. **A–B** SDS-PAGE followed by Western Blotting shows that densitometric ratio of AKTP with β -actin is decreased in AE treated cells for24 hour. Results are presented as Means ± SEM of 4 independent *in vitro* experiments. **C–D.** Decreased immuno-expression of AKTP in xenograft tumors after being treated with AE (400 μg/mL) for 3 weeks. **D.** The histogram shows the percentage of AKTP immuno-positive cells compared to total cells in control and AE treated groups. Data are means ± S.E.M. of 5 different mice. * indicates P<0.05 compared with control group. Bar=25 μm.

Previous experiments showed that AE treatment increased IGF1R expression in SKOV3 cells and also in mouse xenograft tumors. IGF1R activates the AKT signaling pathway [15]. Ectopic insertion of miR-375 resulted in a significant reduction of IGF1R expression and its downstream signaling molecule AKT at both mRNA and protein levels in other cancer cell lines [15]. MiR-375 suppresses malignant behavior of other cancer cells through the AKT signaling pathway [28]. Therefore, the effect of AE on Akt phosphorylation both *in vitro* and in the mouse xenograft tumor was studied. Results summarized in Figure 8A–8D show that AE treatment significantly reduced the levels of AKTP in SKOV3 cells and in mouse xenograft (P≤0.02).

## DISCUSSION

Principal findings of these studies show that AE caused more than 2000-fold increase in miR-375 expression and down regulated the expression of IGF1R and SNAIL1 in OC cells. AE downregulated the expression of IGF1R and SNAIL1 in a mouse model of xenograft OC as well. The functional consequence of decreased SNAIL1 expression was corroborated by upregulation of E-cadherin that is negatively controlled by SNAIL1. AE attenuated xenograft tumor growth *in vivo* as well as inhibited cell proliferation, migration and invasiveness of SKOV3 *in vitro*. AE appears to modulate the effect of OC cells on tumor microenvironment as shown by an increased release of exosomes containing miR-375 from SKOV3 cells. Collectively, these novel results demonstrate that AE affects intracellular and extracellular processes that promote OC growth and metastasis.

Medicinally valuable plant-derived preparations may target multiple cellular sites through a naturally occurring mix of compounds. We believe such products with low toxicity may strengthen our approach to cancer treatment. AE is known to contain a number of active components that may directly influence cellular processes at the gene level [5]. However, classically most studies have focused on frequently reported anti-oxidant and anti-inflammatory mechanisms [5]. Recent reports show that polyphenols in phytomedicinal products modulate microRNA expression in cancer cells [29]. The effects of AE on gene expression, transcription and translation in cellular pathobiology are not well-understood.

Cellular proliferation, migration, invasion and metastasis are key features of tumors aggressiveness [30]. In addition, cellular mobility is crucial for normal development as well as organogenesis, inflammation and wound healing [31]. Dysregulation of the signaling mechanisms involved in cell migration may contribute to increased invasiveness and metastatic properties of cancer cells [32]. Previously, we demonstrated that AE inhibits proliferation in OVCAR3 ovarian cancer cells [6]. Since proliferation and migration are strongly related and SKOV3 cells demonstrate high migration rates [33], we evaluated the effect of AE on migration of SKOV3 cells. Our results show that AE inhibits proliferation, migration and invasiveness of SKOV3 cells. The crossing of basement membranes by cancer cells is a crucial aspect of metastasis [34] (Figure 3). These observations suggest that AE attenuates ovarian cancer growth and metastasis.

MicroRNAs are being increasingly recognized as specific targets and potential therapeutic agents to treat certain diseases including cancer [35]. These non-coding small molecules with approximately 22 nucleotides influence a wide variety of cellular processes including cell proliferation, migration and tumorigenesis [36]. Altered expression of specific miRNA causes measurable abnormalities by impacting the translation of target genes. MiR-375, expressed in many tissues including the ovary [37], was initially found to play an essential role in pancreatic islet development [38]. Ectopic expression of some miRs (such as miR-218, miR-145) inhibits invasion, angiogenesis, and metastasis [39, 40]. Recently, miR-375 was identified as an important regulator in tumorigenesis and cancer progression [41]. MiR-375 inhibits cancer cell proliferation, invasion, and cell motility [25] and its expression is downregulated in ovarian cancer [37], gastric cancer [42], head and neck squamous cell carcinoma [43], and pharyngeal squamous cell carcinoma [44]. MiR-375 increases the effect of platinum based treatment in OC [37]. These observations suggest that miR-375 may function as a suppressor of certain tumors. Conversely, the expression of miR-375 is upregulated in ERα-positive breast cancer [45], lung adenocarcinoma patients [46] and prostate cancer patients [47]. The pro- or anti-oncogenic effect of miRs is determined by the target protein through miR-mRNA interaction. Thus, some miRs may be pro-oncogenic for one type of cancer but act as suppressors of other types of cancer. We determined the effect of AE on several miRs associated with OC and found a 2000-fold increase in miR-375 expression in SKOV3 ovarian cancer cells within 48 hours.

MiR-375 regulates the expression of several target proteins including IGF1R [15]. Epigenetic silencing of miR-375 causes the upregulation of IGF1R [48]. We selected IGF1R as a representative target since it mediates proliferation, differentiation and angiogenesis [16]. Present work shows a strong attenuation of proliferation of SKOV3 cells by AE (Figure 1A). These results suggest that AE may block angiogenesis *via* upregulating miR-375 which, in turn, downregulates IGF1R.

IGF1R belongs to a group of receptors that maintain the AKT-mTOR signaling pathway and thereby influence growth and proliferation [49]. IGF1R is directly involved in the activation of AKT [16] through phosphorylation and AKTP mediates signaling for proliferation and growth [50]. Furthermore, AKT promotes tumorigenesis [51]. Decreased AKTP in AE treated cells suggests that downregulation of AKT phosphorylation by miR-375 mediates the effect of AE. This supports our previous finding of upregulation of autophagy by AE in OC cells [6]. These results suggest that AE may induce autophagy *via* downregulation of AKT phosphorylation.

Micro RNAs are known to play an integral role in modulating epithelial to mesenchymal transition (EMT) during tumorigenesis and metastasis [21]. SNAIL1 is a transcriptional repressor of E-cadherin in epithelial cancer cells and downregulates miR-375 [52]. SNAIL1, in turn, is targeted by miR such as miR-182, miR-30, miR-1, miR-29b, miR-34, miR-203 [53]. E-cadherin, a calcium dependent cell adhesion molecule, plays an important role in the growth and development of cells *via* controlling tissue architecture and maintaining tissue integrity. Loss of expression or function of E-cadherin marks local invasion of epithelial tumor cells and promotes tumor progression [54]. E-cadherin expression is low in ascitic fluid from ovarian cancer and at metastatic sites [54]. Ovarian cancer cells with low E-cadherin expression are more invasive [54] and low E-cadherin predicts poor survival rate compared with ovarian cancer patients expressing high E-cadherin [55]. In the present study we found that AE treatment increased miR-375 and E-cadherin but decreased SNAIL1 expression in SKOV3 cells and in the mouse xenograft. AE-induced increase in the expressions of miR-375 and E-cadherin may attenuate EMT by targeting SNAIL1 in SKOV3 cells. The interaction between miR-375 and SNAIL1 is a subject for separate future studies.

OC is a highly metastatic cancer where the inhibitory effect of AE on the transformation and/or migration of cancer cells may have significant implications for the interaction between cancer cells and tumor microenvironment. Exosomes are now considered as effective mediators of communication between cancer cells and their environment [12]. Exosomes are small secreted membrane vesicles (30-100 nm) that contain miRs and a variety of cell surface and cytoplasmic proteins as their cargo. Exosomes are believed to influence diverse biological processes including angiogenesis, proliferation, tumor cell invasion and metastasis, immune response, and antigen presentation through transfer of proteins, mRNAs and non-coding RNAs to neighbouring or distant cells [56]. Cancer cell-derived exosomes contribute to the recruitment and reprogramming of cells and molecules associated with tumor environment [57]. The effect of AE on exosomes derived from OC cells is not known. Present data demonstrate that AE increases intracellular miR-375 and induces the release of exosomal miR-375. Thus, these results suggest that miR-375 released from AE-treated OC cells modifies tumor microenvironment to attenuate cancer progression.

**Figure 9:**
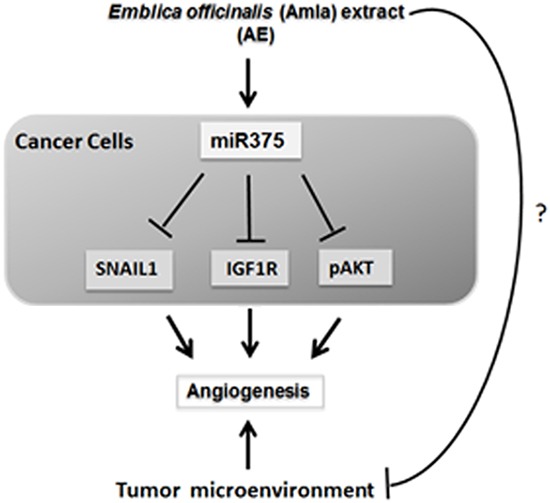
Schematic of the proposed mechanism of action of *E. officinalis* on angiogenesis in OC

The release of miR-375 in exosomes, the down regulation of IGF1R, SNAIL1 and upregulation of E-cadherin suggest a likely effect of AE on the interaction between cancer cells and their environment. Angiogenesis is a continuous parallel process required for tumor growth. We have previously found that AE attenuates angiogenesis [6]. Together, these observations suggest that AE reduces angiogenesis in ovarian cancer through modulation of multiple targets. This novel finding provides a rationale for confirming the effect of AE in multiple cell lines. A number of cell lines of OC with well-identified anatomical origins are known. These include cells from high grade serous tumor (e.g., TOV2978G), high grade serous ascites (OV4453), adenocarcinomas (e.g. OVCAR3), serous adenocarcinomas (e.g., OVCAR4), low grade serous ascites (VOA1312_CL), other specific cell lines including snu8, and UWB1.289 (BRCA1 null). Some of these lines are considered more aggressive and others less aggressive than SKOV3 cells. Confirmation of the cellular effects of AE using multiple cells lines will provide a basis for validating multiple targets of AE. Toward these goals, we propose that AE targets multiple sites that alter tumor microenvironment that inhibit angiogenesis leading to interference with tumor physiology and growth. Figure 9 summarizes our findings and highlights the questions that we plan to address in future studies.

In conclusion, this study indicates for the first time that AE inhibits growth of OC cells *in vitro* and *in vivo*, perhaps through the activation of miR-375 and by targeting the pro-angiogenic IGF1R and SNAIL1. Traditional use of *E. officinalis* in many cultures, the amount of AE (400 μg/mL) needed to observe its effect *in vitro* and the tumor suppressive effect of the dose used in our studies (100 mg/kg body weight/day) suggest feasibility of testing *E. officinalis* in future studies on human subjects. Present results provide a potential mechanistic basis for the multi-target effect of a traditionally used plant-derived product.

## MATERIALS AND METHODS

### Ethics statement

Research described hereunder was conducted in agreement with the ethical standards according to the Declaration of Helsinki, National and International Guidelines, and was approved by the Institutional Review Board, Kansas City VA Medical Center, Kansas City, MO.

### Cell culture and treatment

SKOV3 cells were used as representative OC cells for the present studies. These cells are known to proliferate aggressively and high invasiveness [14, 58]. Epithelial OC cells (SKOV3) and normal placental cells (Hs 799.Pl, ATCC CRL 7530) from American Type Culture Collection (ATCC) were grown in DMEM (Sigma-Aldrich, St. Louis, MO) containing 10% fetal bovine serum (FBS) and incubated at 37°C for 24 h prior to experiments. The medium was replaced with DMEM containing FBS and AE (100-1000 μg/mL) in quadruplicate for 24 h at 37°C. The *in vitro* control group of cells received vehicle (sterile saline). AE for treatment was prepared as reported previously [6].

### Cell proliferation/viability assay

Cell proliferation/viability was measured using [3-(4,5-dimethylthiazol-2-yl)-2,5-diphenyltetrazolium bromide)] (MTT) assay as described previously [59]. SKOV3 cells (5000 cells/well), grown in 96-well plates, were treated with AE (0-1000 μg/mL) for 6-96 h followed by incubation with MTT (0.1 mg/well) in phenol red-free DMEM for 4 h at 37°C. Cells were incubated with isopropanol to dissolve the blue formazon product. Optical density (OD) was measured at 560 nM in a Synergy H1 plate reader (Biotek, Winooski, VT, USA). Number of functionally active cells was determined from optical density values for comparison between control and treated groups from 6 independent experiments performed in duplicates.

### Lactate dehydrogenase (LDH) cytotoxicity assay

Potential necrosis (plasma membrane damage or rupture) in AE-treated SKOV3 cells and normal placental cells (HS 799.Pl) by AE was assessed using Pierce LDH cytotoxicity assay kit (Thermo Fisher Scientific, Waltham, MA USA). Cells (~8000 cells/well) were grown in a 96-well plate. After treatment with AE for 24, 48 and 96 h, 50 mL aliquots of the medium were collected and LDH assay performed according to the manufacturer's instructions. Cytotoxicity was determined from the absorbance measured at 490 nm from six independent experiments performed in duplicates.

### Wound healing assay

The *in vitro* scratch assay is a well-developed method to measure cell migration *in vitro* [60]. Scratch wounds were induced to 70–80% confluent SKOV3 cells, treated with 0 or 400 μg/mL AE and scratched areas were photographed at 0 and 24 h using a phase contrast inverted microscope (Axiovert 100 TV, Carl Zeiss AG, Oberkochen, Germany). The relative migration gap distance was calculated by the following formula: the relative migration distance (%) = 100 (A–B)/A, where A and B are the widths of wound area before and after incubation respectively.

### Invasion assay

The transwell invasion assay was performed as described previously [61]. Insert wells of 24-well Transwell system (Corning®, NY, USA) were coated with Matrigel (100 μL/well) in 0.7% NaCl (0.01M Tris-HCl, pH 8.0). Cells (7×10^4^/well) in serum-free medium (250 μL) were layered on each insert. AE (0-1000 μg/mL) in medium (600 μL/well) containing 10% FBS was added to wells. At 24 h, inserts were fixed in cold 100% methanol for 20 min, stained with 0.1% crystal violet in 20% methanol for 10 min at room temperature and washed with deionized water. Cells remaining on the upper side of the membranes were removed with cotton swabs and inserts were dried overnight in dark. Stained membranes were photographed in three random non-overlapping fields using a digital photomicroscope (Leica Microsystems, Buffalo Grove, IL, USA).

For quantitative analysis of cell migration, inserts were covered with 300 μL of 10% acetic acid and optical density (OD) of extracted crystal violet was measured at 590 nm using a microplate scanning spectrophotometer (BioTek, Winooski, VT, USA). Cell-free inserts containing only medium were used as OD background controls. Results are presented as average background (OD) - experimental (OD) ± SEM obtained from three independent experiments performed in duplicates.

### Immunostaining of cultured cells

Immunocytochemistry was performed using previously described methods [6]. SKOV3 cells were fixed in 4% buffered formaldehyde and washed in phosphate buffered saline (PBS), treated with blocking serum (ImmPRESS, Vector Laboratories, Burlingame, CA) and incubated with antibodies to SNAIL1, E-cadherin (Cell Signaling, Boston, MA) or IGF1R (EMD Millipore, Billerica, MA) (1:100 dilution) overnight at 4°C. Cells were washed and immunoreactivity was detected using streptavidin-conjugated secondary antibodies (ImmPRESS, Vector Laboratories) and counterstained with hematoxylin. A Leica digital microscope was used for imaging to determine the percentage of immunostained cells (immunostained cells/total number of cells x 100).

### Immuno-staining of tissue sections

Immunohistochemistry was performed using previously described methods [6]. Briefly, tissue sections fixed in 4% buffered formaldehyde were deparaffinized, rehydrated in descending concentrations of alcohol, washed with PBS and treated with blocking serum. Tissue slices were incubated with SNAIL1, E-cadherin (1:100), IGF1R (1:500) and AKTP (1:400, Cell Signaling) antibodies overnight in a humid chamber at 4°C. Hematoxylin was used as a counterstain and a digital microscope (Leica Microsystems) to image the stained sections.

### Western blot analysis

Western blotting was performed as previously described [6]. Briefly, total protein concentration in the cell lysate was determined by using DC™ protein assay (Bio-Rad Laboratories, Hercules, CA). Fifty micrograms of total protein were analyzed using sodium dodecyl sulfate-polyacrylamide gel electrophoresis (SDS-PAGE) and electro-transferred to a nitrocellulose membrane. The membrane was incubated with blocking buffer (Thermo Fisher) for 1 h at room temperature and incubated overnight at 4°C with the primary antibody to SNAIL1, E-cadherin (1:1000 dilution, Cell Signaling), IGF1R (1:1000 dilution, Abcam, Cambridge, MA, USA), CD9, CD63 or HSP70 (1:1000 dilution, SBI System Biosciences, Mountain View, CA). The membrane was washed with Tris-buffered saline-Tween-20 buffer (TBST) and incubated with HRP-conjugated secondary antibodies (1:10,000 dilution, Abcam) at room temperature for 1h. After washing with TBST immunoreactivity was detected using enhanced chemiluminescence reagent (GE Healthcare Bio-Sciences, Marlborough, MA). β -actin, the loading control, was detected using β -actin antibody (1:10000, Abcam) and anti-mouse IGG (1:10000, Sigma Aldrich) as the primary and secondary antibodies, respectively.

### Transfection

SKOV3 cells (3×10^5^) at 70% confluency were divided into untreated control, miR-375 inhibitor-treated and miR-negative control groups. Some cells were transfected with Anti-hsa-miR-375 miScript miR Inhibitor (MIMAT0000728: 5′UUUGUUCGUUCGGCUCGCGUGA3′, 2 μg; Life Technologies, Thermo Fisher Scientific, Carlsbad, CA) (miR-375 inhibitor). At 6 h, the medium was replaced with fresh high-glucose DMEM containing 10% FBS. At 48 h, green fluorescence was observed under an inverted fluorescence microscope (Carl Zeiss) for the preliminary analysis of transfection efficiency. To study the effect of AE, miR-375 inhibitor-transfected cells after 24 h of transfection were incubated with AE for additional 24 h. The cells were then processed to determine the expression of the genes and proteins.

### RNA extraction

Total RNA from SKOV3 cells and tumor samples was extracted using the RNAzol RNA/miRNA Isolation Kit following the manufacture's protocol (Molecular Research Center, Cincinnati, OH, USA). The quantity and quality of mRNA were determined by measuring absorbance using microwell plate reader (Biotek) and by RNA Experion Chip (BioRad Laboratories, Hercules, CA, USA).

### MicroRNA analysis

Total RNA was isolated from control and AE-treated SKOV3 cells by RNAzol method as described in the previous section. cDNA was synthesized using TaqMan™ Advanced miRNA cDNA Synthesis Kit (Life Technologies-Applied Biosystems, Foster City, CA).

### Real time RT-PCR (RT-qPCR)

Total RNA (1.5 mg/sample) was reverse transcribed using the iScript™ cDNA Synthesis kit according to manufacturer's instructions (BioRad). The primers were designed using the Primer3 software (MIT) and purchased from Integrated DNA Technologies Inc., Coralville, Iowa, USA). The sequences of forward and reverse primers were:

- human 18S (GenBank Accession no. NM_002046) 5′CTCTCTGCTCCTCCTGTTCGAC3′ and 5′GAGC GATGTGGCTCGGCT3′
- Human RPL4 (GenBank Accession no. NM_000 968.3) 5′CGCTTCCCTCAAGAGTAACT3′ and 5′CT CTTTGGATCTCTGGGCT3′
- Human IGF1R (GenBank Accession no. NM_000 875.4) 5′CGACATCCGCAACGACTATC3′ and 5′AC AGCAGCAAGTACTCGGTA3*′*
- Human SNAIL1 (GenBank Accession no. NM_0059 85.3) 5′CCAGACCCACTCAGATGTC3′ and 5′GACT CTTGGTGCTTGTGGA3′

The primers for microRNAs (miR-375 - catalog # 000564; miR27a - catalog # 000408; miR-195a - catalog # 000494; miR-10b - catalog # 002218; miR-let7a - catalog # 000377; miR-let7c - catalog # 000379; miR-146a - catalog # 000468 and RNU 58A - catalog # 001207) were purchased from Life Technologies. Real-time qPCR (RT-qPCR) was performed using Fast SYBR Green on a Real-Time PCR System (BioRad). Relative expression values were calculated using the 2 (−^DD^C (T) method and were normalized against reference genes 18S and RPL4. The specificity of amplification was confirmed by evaluation of the melting curves.

### Exosome isolation

SKOV3 cultures were treated with AE in FBS-free DMEM for 48 h. Aliquots of the medium were collected to isolate exosomes using ExoQuick-TC™ following the manufacturer's instructions (SBI System Biosciences, Mountain View, CA). Exosomal RNA was extracted using miRCURYTM RNA isolation kit according to the manufacturer's protocol (Exiqon, Weburn, MA). Exosomal proteins were extracted using RIPA buffer containing protease inhibitors. Presence of exosomal marker proteins CD9, CD63 and HSP70 was confirmed by Western blotting.

### Xenograft tumor studies in athymic nude mice

Animal care and experimental procedures were performed following protocols approved by the Institutional Animal Care and Use Committee (IACUC) and the R&D Committee, Kansas City VA Medical Center (KC VAMC), Kansas City, MO.Nude mice (nu/nu genotype, Harlan Laboratories, Madison, WI) were maintained at the Animal Resource Facility, KC VAMC in a pathogen-free environment with *ad libitum* water and food in a 12 h light and 12 h dark cycle. Eight weeks old mice were injected subcutaneously with SKOV3 cells (5×10^6^) with Matrigel into the right rear flank (5 mice per group) and treated with AE (100 mg/kg body weight/day in 10% sucrose) for 3 weeks as described previously [6]. The untreated control group received 10% sucrose solution. Tumor volume was measured twice/week for 3 weeks. Mice were sacrificed at the end of the treatment period, tumors were removed and processed for further analysis.

### Statistical analysis

Assays were accomplished in duplicates and each experiment was repeated at least thrice or as described. All graphical data are displayed as mean ± S.E.M. Significance was tested using unpaired, two-tailed Student's t-Test with unequal variance (Microsoft Excel, Redmond, WA) and one-way analysis of variance (ANOVA). P≤ 0.05 was considered significant.
